# Rab32 and Rab38 maintain bone homeostasis by regulating intracellular traffic in osteoclasts

**DOI:** 10.1247/csf.23061

**Published:** 2023-10-04

**Authors:** Kanako Tokuda, Shiou-Ling Lu, Zidi Zhang, Yumiko Kato, Siyu Chen, Kazuya Noda, Katsutoshi Hirose, Yu Usami, Narikazu Uzawa, Shinya Murakami, Satoru Toyosawa, Mitsunori Fukuda, Ge-Hong Sun-Wada, Yoh Wada, Takeshi Noda

**Affiliations:** 1 Graduate School of Frontier Biosciences, Osaka University, Osaka 565-0871, Japan; 2 Department of Oral Cellular Biology, Center for Frontier Oral Science, Graduate School of Dentistry, Osaka University, Osaka 565-0871, Japan; 3 Department of Oral & Maxillofacial Oncology and Surgery, Graduate School of Dentistry, Osaka University, Osaka 565-0871, Japan; 4 Department of Periodontology and Regenerative Dentistry, Graduate School of Dentistry, Osaka University, Osaka 565-0871, Japan; 5 Department of Oral and Maxillofacial Pathology, Graduate School of Dentistry, Osaka University, Osaka 565-0871, Japan; 6 Department of Integrative Life Sciences, Graduate School of Life Sciences, Tohoku University, Miyagi 980-8578, Japan; 7 Department of Biochemistry, Faculty of Pharmaceutical Sciences, Doshisha Women’s College, Kyoto 610-0395, Japan; 8 Department of Biological Sciences, Institute of Scientific and Industrial Research, Osaka University, Osaka 565-0871, Japan; 9 Center for Infectious Disease Education and Research, Osaka University, Osaka 565-0871, Japan

**Keywords:** Rab32, Rab38, osteoclast, lysosome-related organelle, secretory lysosome

## Abstract

Osteoclasts play a crucial role in bone homeostasis by forming resorption pits on bone surfaces, resulting in bone resorption. The osteoclast expression of Rab38 protein is highly induced during differentiation from macrophages. Here we generated mice with double knockout (DKO) of Rab38 and its paralogue, Rab32, to investigate the roles of these proteins in osteoclasts. Bone marrow-derived macrophages from Rab32/38 DKO mice differentiated normally into osteoclasts *in vitro*. However, DKO osteoclasts showed reduced bone resorption activity. These osteoclasts also demonstrated defective secretion of tartrate-resistant acid phosphatase and cathepsin K into culture medium. Furthermore, the plasma membrane localization of *a*3, an osteoclast-specific *a* subunit of V-ATPase, was abrogated in DKO mice, substantiating the reduced resorption activity. *In vivo*, Rab32- and Rab38-positive cells were attached to the bone surface. Eight-week-old DKO mice showed significantly thickened trabecular bones in micro-CT and histomorphometry analysis, as well as reduced serum levels of cross-linked C-telopeptide of type I collagen, indicating diminished bone resorption *in vivo*. In DKO male mice over 10 weeks of age, hyperostosis appeared at the talofibular syndesmosis, the distal junction of the tibia and fibula. Furthermore, middle-aged mice (10 to 12 months of age) exhibited kyphosis, which is not usually observed in wild-type male mice until around 24 months of age. These results indicate that Rab32 and Rab38 contribute to osteoclast function by supporting intracellular traffic, thereby maintaining normal bone homeostasis.

## Introduction

Bone homeostasis, including bone structure and function, is maintained by a delicate balance between bone formation by osteoblasts and bone resorption by osteoclasts (Boyle *et al.*, 2003). Accordingly, a number of bone-related diseases are associated with osteoclast malfunction: excessive activation of osteoclasts leads to diseases such as osteoporosis, periodontitis, and rheumatoid arthritis, whereas suppression of osteoclast activity and bone resorption leads to conditions involving bone sclerosis, such as marble bone disease (Rodan and Martin, 2000; Teitelbaum and Ross, 2003). Osteoclasts are multinucleated giant cells that originate from the intercellular fusion of monocyte-macrophage lineage cells (Boyle *et al.*, 2003). Osteoclasts differentiate upon stimulation by macrophage colony-stimulating factor (M-CSF) and receptor activator of NF-κB ligand (RANKL), which are paracrine factors produced by osteoblasts and osteocytes (Boyle *et al.*, 2003). Osteoclasts adhere to the bone surface and form a zone termed the ruffled border, characterized by a wavy area of the plasma membrane (Stenbeck, 2002). Osteoclasts secrete H^+^ and hydrolytic enzymes into the ruffled border, whose periphery is sealed by an actin ring to form a bone resorption pit (Coxon and Taylor, 2008). The ruffled border and resorption pit are together functionally equivalent to lysosomes in that they contain vacuolar H^+^-ATPase (V-ATPase) and hydrolytic enzymes such as cathepsin K and tartrate-resistant acid phosphatase (TRAP), and thereby digest bone matrix in the same way that lysosomes digest incorporated materials (Lacombe *et al.*, 2013; Stenbeck, 2002). It has also been reported that cathepsin K and TRAP accumulate within secretory lysosomes in organelles, and that these organelles fuse with the plasma membrane to form active bone resorption pits (van Meel *et al.*, 2011). Although secretory lysosomes require further characterization, they may be considered to be a type of lysosome-related organelle (LRO), defined as a specialized organelle possessing lysosome-like properties and cell type-specific functions (Coxon and Taylor, 2008; Luzio *et al.*, 2014). An example of an LRO is the melanosome, which stores melanin pigments in melanocytes (Delevoye *et al.*, 2019; Luzio *et al.*, 2014).

Rab is a class of small GTPase-family proteins that are broadly conserved in eukaryotes and that function as fundamental regulator of membrane traffic (Pfeffer, 2017). Mammals contain approximately 60 different Rab proteins that are assumed to play specialized roles in various cellular processes (Homma *et al.*, 2021). Rab32 and Rab38 are close paralogues that are specifically expressed in LRO-containing tissues and cells such as melanocytes and platelets (Ohbayashi *et al.*, 2017). The two proteins have overlapping functions in melanosome biogenesis in melanocytes (Wasmeier *et al.*, 2006). Both localize on the melanosome membrane and regulate post-Golgi vesicular transport of melanosomal enzymes to immature melanosomes (Tamura *et al.*, 2011, 2009; Wasmeier *et al.*, 2006). *Chocolate* mice possess a point mutation in Rab38 and show typical characteristics of LRO deficiency, such as impaired melanosome biosynthesis resulting in coat color depigmentation, and pronounced expansion of the lamellar bodies due to abnormalities in surfactant secretion in the lungs (Oiso *et al.*, 2004; Osanai *et al.*, 2008). Rab38 is the gene responsible for Hermansky-Pudlak syndrome (HPS), in which defective biogenesis of LROs results in an abnormal pulmonary phenotype, predisposition to hemorrhage, and oculocutaneous albinism (Huizing *et al.*, 2000). A mouse model of severe HPS involving Rab32/38 double knockout (DKO) is characterized by an atypical coat color, eye pigment dilution, and abnormal lung morphology with reduced numbers of platelet dense granules (DGs) (Aguilar *et al.*, 2019). In human platelets, single deletion of Rab38 or Rab32 does not affect DG biosynthesis or function, indicating that Rab32 and Rab38 play redundant roles in the biosynthesis of DGs (Aguilar *et al.*, 2019).

We recently revealed that Rab38 expression is highly induced during osteoclast differentiation (Noda *et al.*, 2023). NFATc1 is a master regulatory transcription factor that regulates osteoclast differentiation (Takayanagi *et al.*, 2002), and it has been reported that Rab38 is upregulated in an NFATc1-dependent manner in osteoclasts (Charles *et al.*, 2012). We also reported that Rab32 and Rab38 co-localize on LROs in osteoclasts (Noda *et al.*, 2023). In this study, we investigate the role of Rab32 and Rab38 in osteoclast function. We generated Rab32/38 DKO mice and evaluated the effects on bone resorption and structural changes.

## Results

### Establishment of Rab32/38 DKO mice

**Fig. 1:**
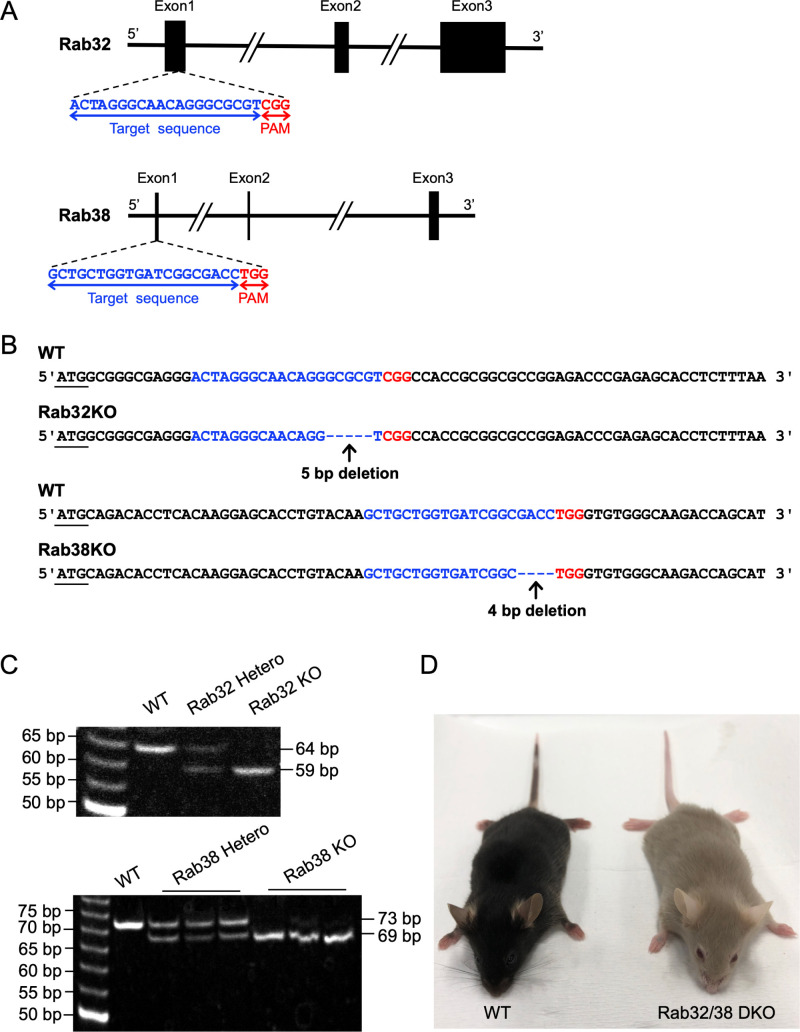
Establishment of Rab32/38 DKO mice A: Schematic of the genome loci of Rab32 and Rab38 with the respective target sites of CRISPR/Cas9. Guide RNAs were designed to target the exon 1 loci for both Rab32 and Rab38. Target sites are shown in blue, and protospacer adjacent motif (PAM) sequences are shown in red. B: Targeted genome sequences of Rab32 and Rab38 in WT and Rab32/38DKO mice. Rab32 KO are deleted of 5 base pairs and Rab38 KO are deleted of 4 base pairs, respectively. Initiation codons were underlined. C: Genotyping of Rab32 and Rab38 WT and KO alleles in WT and hetero and homo mutant mice by PCR. D: Appearance of WT and Rab32/38 DKO mice. Coat color is light beige and eye color is red in the Rab32/38 DKO mouse.

To investigate the role of Rab32 and Rab38 in osteoclast function, Rab32/38 DKO mice were generated from C57BL6/J mice using the CRISPR/Cas9 system. Guide RNAs were designed to target the Rab32 exon 1 and Rab38 exon 1 loci (Fig. 1A). F_0_ mosaic mutant mice were crossed with C57BL/6J mice to produce F_1_ progenies heterozygous for a 4-nucleotide base-pair deletion in Rab32 and a 5-nucleotide base-pair deletion in Rab38. The mutations were confirmed by DNA sequencing, and they were considered to be null alleles because they cause frame-shifts of the reading frames encoded in exon 1 (Fig. 1B). Each offspring was genotyped by specific PCR of their genome (Fig. 1C). The resulting F_1_ heterozygous mutant mice were mated with WT mice to obtain F_2_ heterozygous mutant mice. Furthermore, F_1_ heterozygous mutant mice were mated with F_2_ heterozygous mutant mice to generate F_3_ homozygous mutant mice. Thereafter, homozygous mutant mice were mated with each other after 8 weeks of age to maintain the Rab32/38 DKO mouse line.

Of note, DKO mice exhibited a light beige coat and red eyes, compared to the black coat and black eyes of C57BL6/J wild-type mice (Fig. 1D). This is consistent with the recently reported phenotype of Rab32/38 DKO mice (Aguilar *et al.*, 2019). While Rab32 single knockout mice showed no apparent difference compared to wild-type mice in either coat or eye color, Rab38 single knockout mouse did have a slight pigment deficiency in coats that was also seen in a *chocolate* mouse mutant with a Rab38 natural point mutation (Loftus *et al.*, 2002) (J-STAGE Data). These phenotypes were consistent with previous reports showing crucial overlapping roles of Rab32/38 in melanosome biogenesis (Aguilar *et al.*, 2019; Wasmeier *et al.*, 2006), and supported the successful establishment of the Rab32/38 mutant mouse line.

### Rab32/38 DKO did not affect the differentiation efficiency of bone marrow-derived macrophages into osteoclasts

**Fig. 2:**
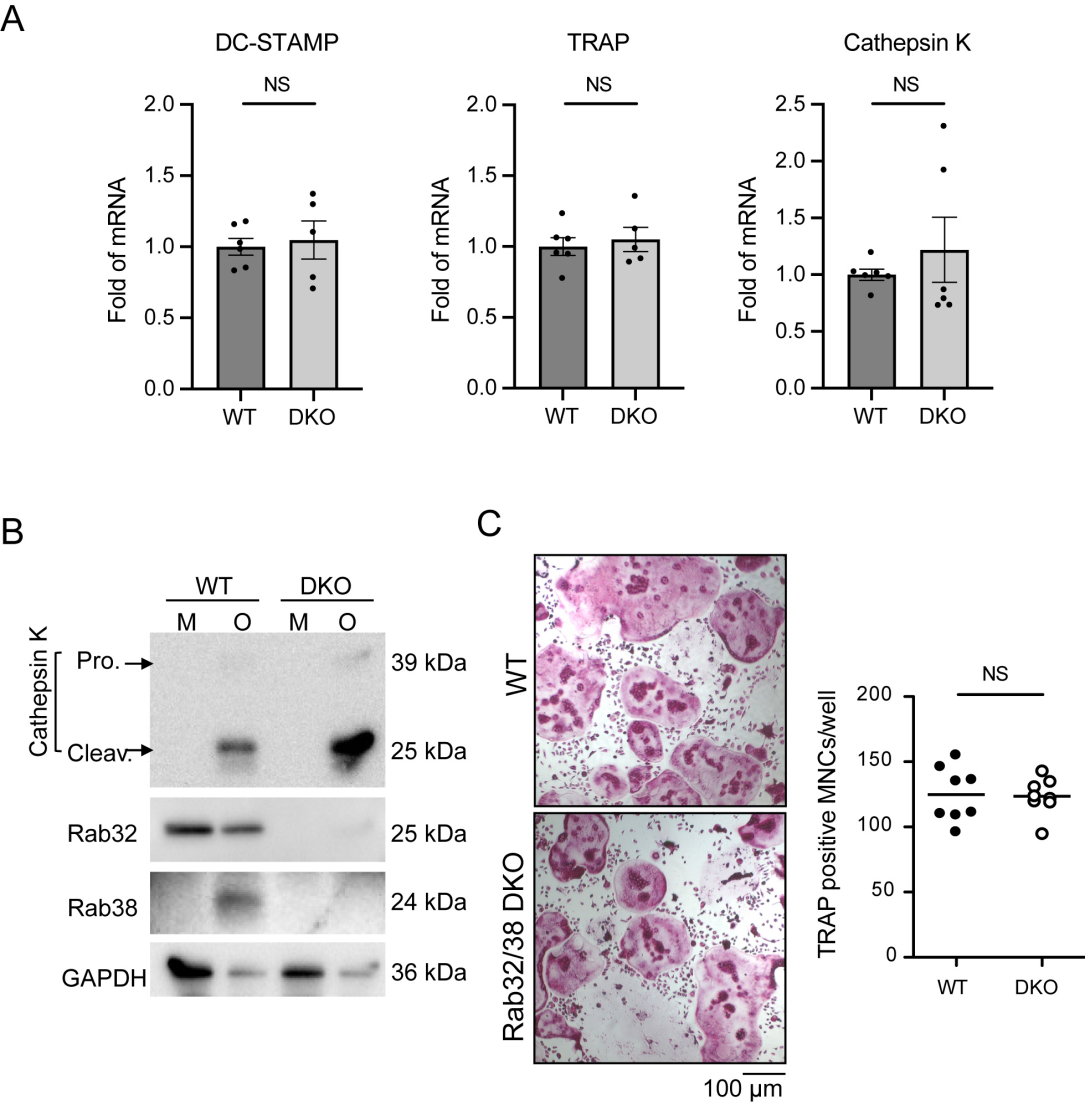
Rab32/38 DKO had no effect on osteoclast differentiation A: BMMs of WT and DKO mice were cultured in differentiation medium for 5–6 days. mRNA expression levels of each marker gene before and after cell differentiation were analyzed by qPCR. Data were normalized using GAPDH as an internal control. The mRNA level of each osteoclast marker gene was normalized to its level in BMMs, and then the fold mRNA induction was normalized to the WT value. Results were obtained in three independent experiments with two technical replicates per PCR reaction. B: Cell extracts of BMMs (M) and osteoclasts (O) from WT and Rab32/38 DKO mice were subjected to western blotting with the indicated antibodies. C: Quantification of TRAP-positive multinucleated cells (MNCs). After induction of differentiation of BMMs from WT and DKO mice for 5 days, TRAP staining was performed. TRAP-positive MNCs (>3 nuclei) in each view field of microscopic images were counted (n = 8). Each data point is indicated as mean ± SD. Statistical analysis was performed using the unpaired Student’s *t*-test (NS = not significant). Scale bar: 100 μm.

We next evaluated the effect of Rab32/38 DKO on bone marrow-derived macrophages (BMMs) differentiation into osteoclasts. Bone marrow from the femurs and tibias of WT and Rab32/38 DKO mice was harvested and cultured with M-CSF for 2 days to induce BMMs. These were then cultured with RANKL for 5 or 6 days to induce differentiation into osteoclasts. The mRNA expression of dendritic cell-specific transmembrane protein (DC-STAMP), tartrate-resistant acid phosphatase (TRAP), and cathepsin K (CTSK), which are induced during BMM differentiation into osteoclasts (Minkin, 1982; Saftig *et al.*, 1998; Yagi *et al.*, 2005), were measured by qPCR. The induction levels of these genes in DKO osteoclasts were indistinguishable from those in WT cells (Fig. 2A).

The total intracellular protein extracts from BMMs and osteoclasts derived from WT and Rab32/38 DKO mice were subjected to western blotting. Rab32 protein expression was confirmed in WT BMMs and osteoclasts, whereas Rab38 was absent in WT BMMs (Fig. 2B), as reported in our recently published paper (Noda *et al.*, 2023). Neither Rab32 nor Rab38 was detected in Rab32/38 DKO osteoclasts or BMMs, confirming the establishment of null mutants of Rab32 and Rab38 (Fig. 2B). Furthermore, the protein extracts from mouse tissues, including heart, spleen, lung, liver, and kidney, were subjected to western blotting with anti-Rab32 and -Rab38 antibodies. Rab32 signals were clearly detected in spleen, lung, and liver in WT mice but not in DKO mice (Sup. fig. 1). Rab38 signals were barely detectable in any of these organs, even in WT mice, implying relatively low expression of Rab38 (data not shown). When the protein extracts from BMMs and osteoclasts of WT and Rab32/38 DKO mice were probed with anti-CTSK antibody, the CTSK signal was highly induced in differentiated DKO osteoclasts and in WT osteoclasts (Fig. 2B). These results further indicate that the differentiation efficiency of BMMs into osteoclasts is barely affected in Rab32/38 DKO condition *in vitro*.

Furthermore, we quantified the number of TRAP-positive multinucleated cells (MNCs) with more than three nuclei per cell, which correspond to osteoclasts, after 5-day culture with RANKL. WT and Rab32/38 DKO mice exhibited comparable numbers of MNCs, with no significant difference in the formation of multinucleated osteoclasts (Fig. 2C). Collectively, deletion of Rab32 and Rab38 had negligible effects on differentiation into osteoclasts *in vitro*, including intercellular fusion and multinucleation.

### Osteoclasts from Rab32/38 DKO mice showed reduced bone resorption capacity

**Fig. 3:**
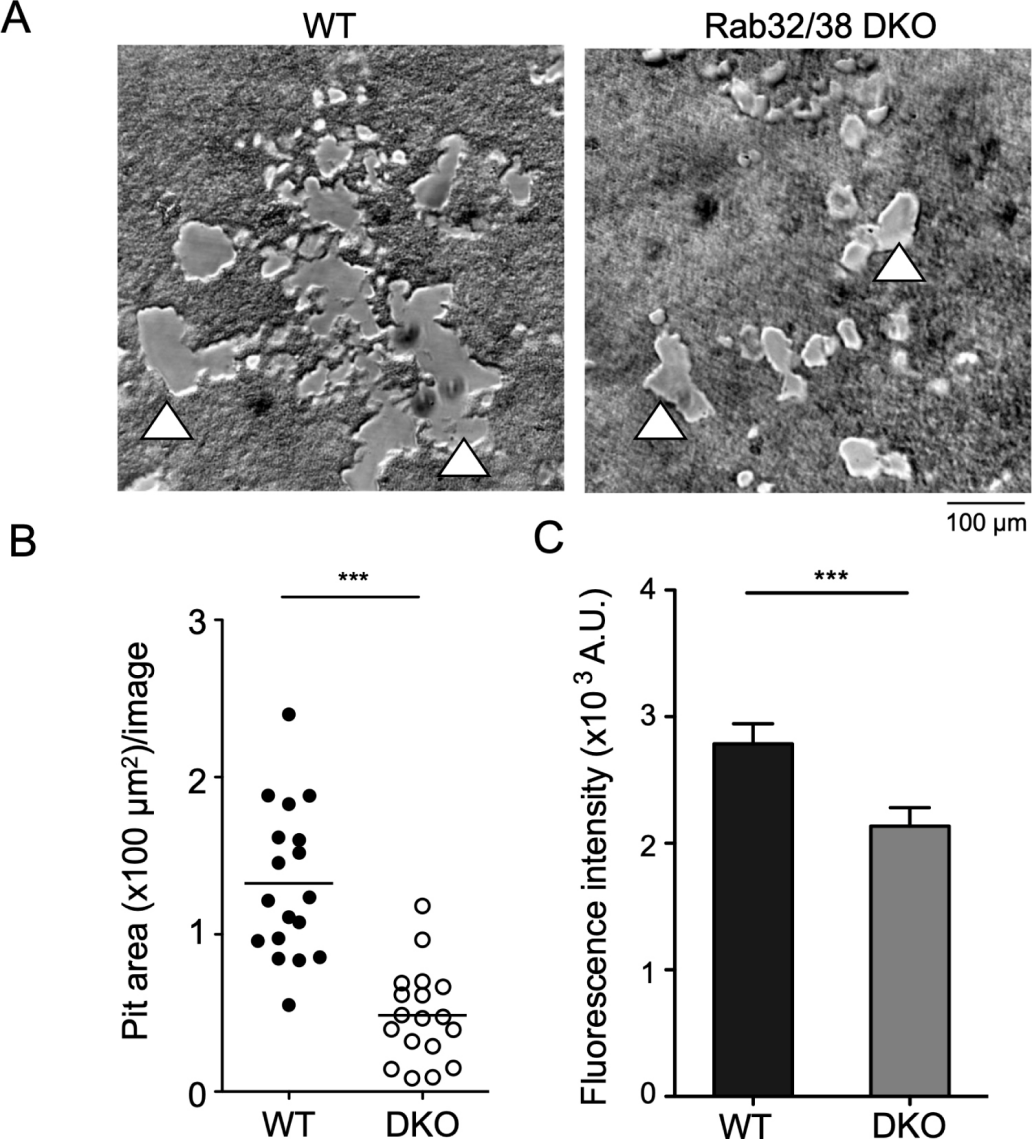
Resorptive capacity of Rab32/38 DKO-derived osteoclast was suppressed A: Resorption pits formed by WT and Rab32/38 DKO-derived osteoclasts. Each osteoclast was cultured on a FACS-labeled calcium phosphate plate for 5 days. Resorbed areas are indicated with triangles. Scale bar: 100 μm. B: The resorption pit area in A was quantified using ImageJ as described in the materials and methods. C: Fluorescence intensity of culture supernatants of WT and Rab32/38 DKO-derived osteoclasts cultured on the FACS-labeled calcium phosphate plate was measured. Each data point is shown as mean ± SD. Statistical analysis was performed using the unpaired Student’s *t*-test (*** *p*<0.001).

We next assessed the bone resorption capacity of osteoclasts derived from Rab32/38 DKO mice. Osteoclasts from WT and DKO mice were cultured for 5 days on culture plates coated with fluoresceinamine-labeled chondroitin sulfate (FACS)-bound calcium phosphate, which was subjected to osteoclast enzyme digestion. The cleavage products released into the medium were detected by fluorescence analysis. The digested pit area was significantly smaller in osteoclasts derived from Rab32/38 DKO mice compared to those from WT mice (Fig. 3A and B). The fluorescence intensity reflecting released FACS was also significantly reduced in Rab32/38 DKO osteoclasts (Fig. 3C), indicating that these cells have significantly reduced bone resorption capacity.

### TRAP and CTSK secretion efficiency were decreased in Rab32/38 DKO osteoclasts

**Fig. 4:**
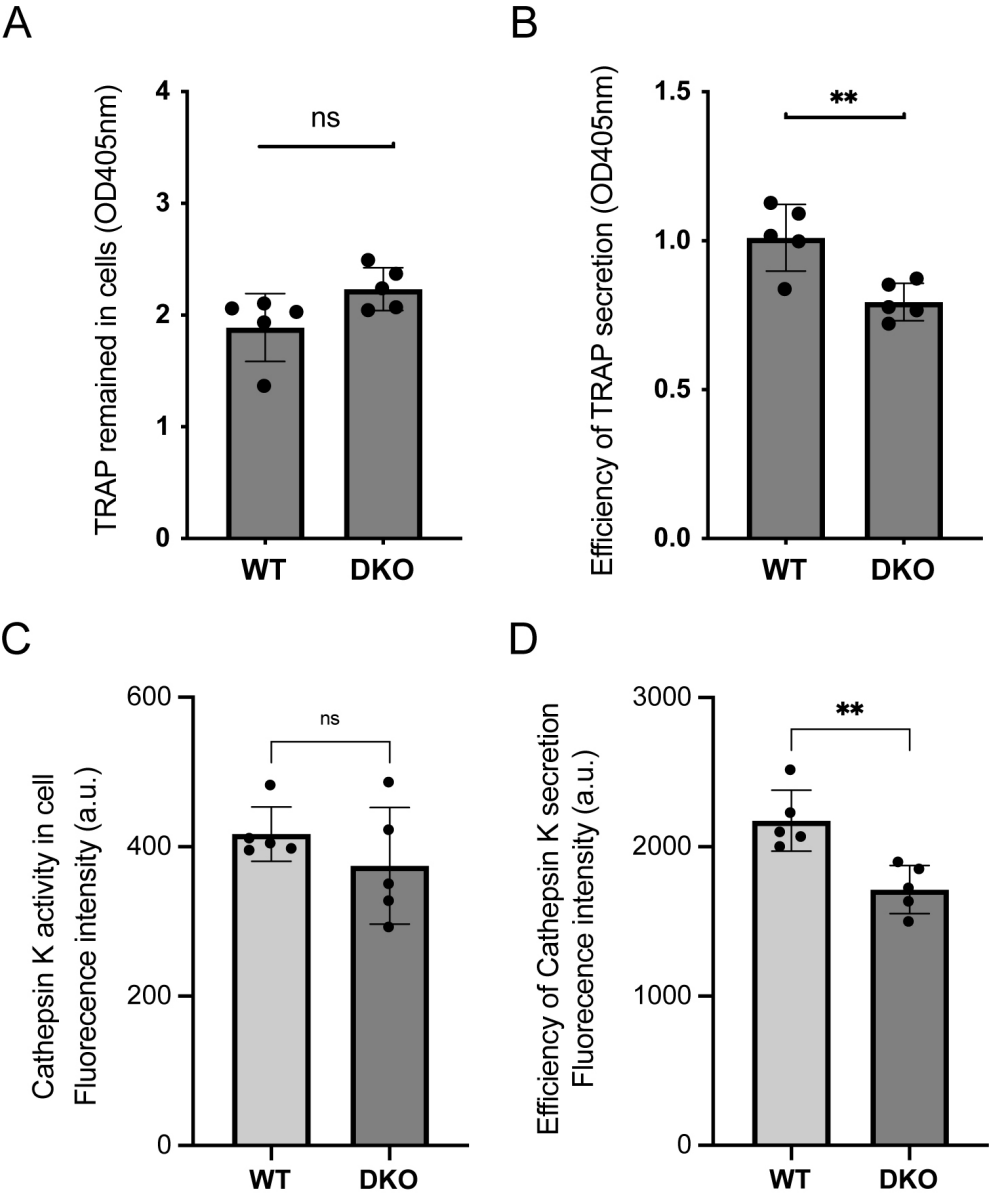
The secretion of TRAP and CTSK was decreased in Rab32/38 DKO osteoclasts A, B: TRAP activity in the intracellular fraction (A) and culture supernatant (B) of osteoclasts derived from WT and Rab32/38 DKO mice, as described in the materials and methods. C, D: CTSK activity in the intracellular fraction (C) and culture supernatant (D) of osteoclasts derived from WT and Rab32/38 DKO mice using the Magic Red Cathepsin K Assay Kit, as described in the materials and methods. Each data point is shown as mean ± SD. The analysis included 5 of 96 wells in each condition for the TRAP assay, and 5 of 24 wells in each condition for the CTSK assay. Statistical analysis was performed using the unpaired Student’s *t*-test (** *p*<0.01).

Various enzymes in osteoclasts, including TRAP and CTSK, are released into the resorption pit via vesicular transport (Coxon and Taylor, 2008). First, we examined the distribution of TRAP in osteoclast culture. Intracellular TRAP activity was comparable between WT and DKO osteoclasts (Fig. 4A), which is consistent with the fact that the two cell types showed similar TRAP mRNA expression and TRAP staining intensity (Fig. 2A and C). However, the extracellular TRAP activity in the culture medium was significantly reduced in Rab32/38 DKO osteoclasts (Fig. 4B).

We next examined the distribution of CTSK using a Magic Red Cathepsin K Assay Kit. Similar to the TRAP results, CTSK activity within osteoclasts was similar between WT and Rab32/38 DKO mice (Fig. 4C), reflecting equivalent CTSK mRNA expression (Fig. 2A) and protein levels (Fig. 2B). By contrast, extracellular CTSK activity in culture medium was decreased in Rab32/38 DKO osteoclasts (Fig. 4D). These results indicate that the secretion of TRAP and CTSK to the extracellular space was hampered in Rab32/38 DKO osteoclasts.

### Rab32 and Rab38 were involved in transport of V-ATPase to the plasma membrane

**Fig. 5:**
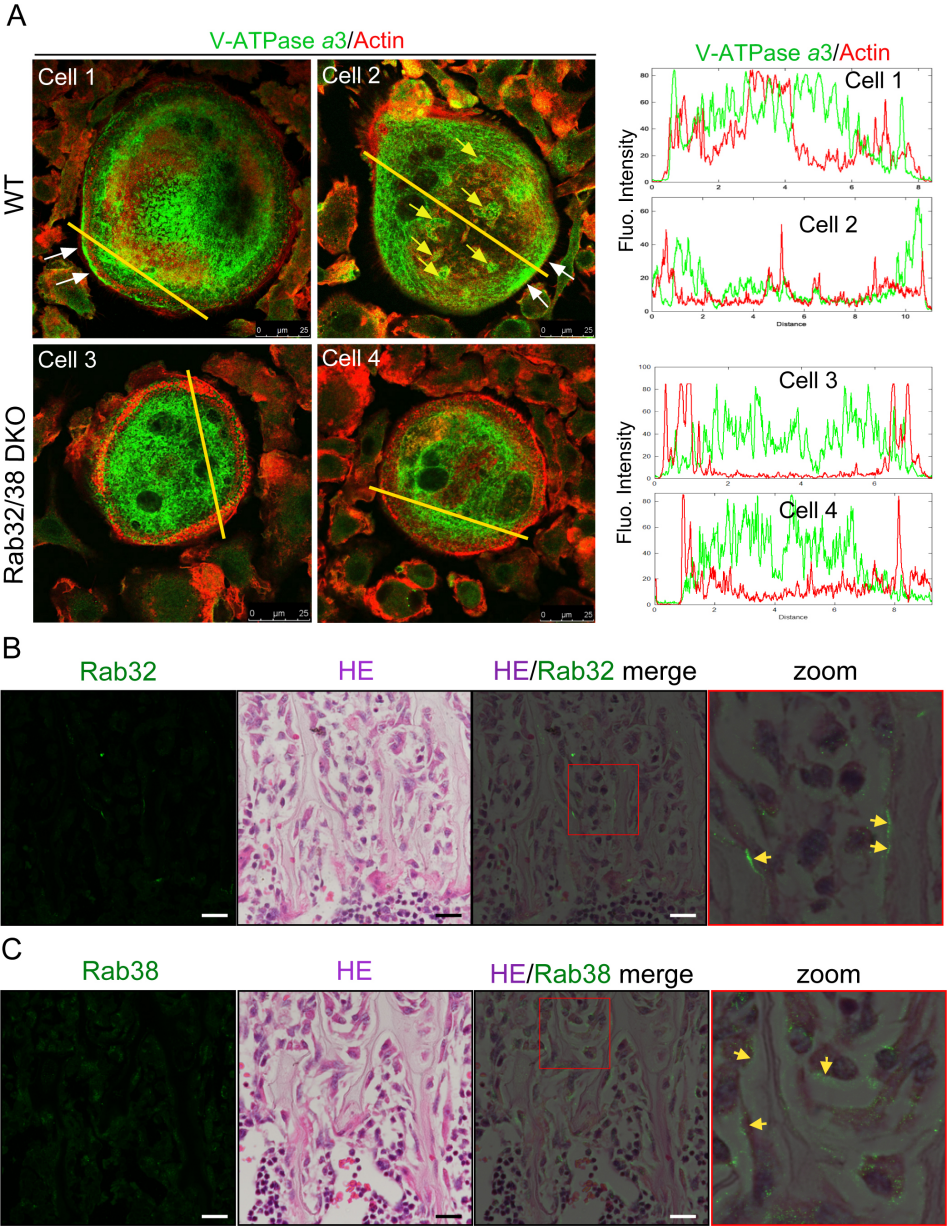
Accumulation of the V-ATPase *a*3 subunit on the plasma membrane was absent in Rab32/38 DKO osteoclasts, and Rab32 and Rab38 localized on bone surface A: Immunofluorescence staining of *a*3 and actin of WT and Rab32/38 DKO osteoclasts. The *a*3 positive plasma membrane is indicated by white arrow, and *a*3-positive ruffled border is indicated by yellow arrow. Scale bar: 25 μm. B: H&E and immunofluorescence staining of mouse bones. Femurs were harvested from 8-week-old male WT mice and processed for section preparation as described in the materials and methods. Sections were stained with anti-Rab32 rabbit or anti-Rab38 rabbit polyclonal antibodies, with Alexa Fluor 488-conjugated anti-rabbit IgG as the secondary antibody. After observation of Rab32 and Rab38 signals with a confocal laser scanning microscope, the specimens were further stained with H&E. Fluorescent and H&E images were superimposed. Scale bar: 20 μm.

In mature osteoclasts, V-ATPase is transported to the ruffled border, where it acidifies the interior of the resorption pit (Nishi and Forgac, 2002; Toei *et al.*, 2010). *a*3 is an isoform of the *a*-subunit of V-ATPase that fulfills this function, and is especially highly expressed in osteoclasts (Sun-Wada *et al.*, 2003; Toyomura *et al.*, 2003). Osteoclasts on a plastic dish form an actin ring adjacent to plasma membranes and express V-ATPase with the *a*3 isoform on their plasma membranes, reflecting the relocalization of the proton pumps to the ruffled border (Toyomura *et al.*, 2003). Immunostaining of WT osteoclasts with anti-*a*3 antibody clearly showed positive staining at the plasma membrane adjacent to the actin ring (61.3%; 27 of 44 actin ring-positive cells) (Fig. 5A; white arrow). In contrast, such staining was markedly reduced in Rab32/38 DKO osteoclasts (18.5%; five of 27 actin ring-positive cells) (Fig. 5A). The frequency of actin ring formation in mature osteoclasts, which contain more than three nuclei in WT mice (88% (44 of 50)), was slightly reduced in DKO mice (60% (27 of 45)). In addition, *a*3-positive ring-like structures and puncta demonstrated intense *a*3 accumulation, which together represented secretory lysosomes (Sup. fig. 2) (Sun-Wada *et al.*, 2003; Toyomura *et al.*, 2003). By contrast, DKO osteoclasts showed few, if any, rings or puncta that were rich in the *a*3 isoform, suggesting that the secretory lysosomes were defective (Sup. fig. 2). Moreover, medium-sized actin rings containing *a*3-positive signals, which could represent small ruffled borders, were occasionally observed in WT osteoclasts but not in DKO osteoclasts (Fig. 5A; yellow arrow). Taken together, these results suggest that Rab32 and Rab38 are involved in the transport of V-ATPase, TRAP, and CTSK to the plasma membrane and to the extracellular space, thus supporting bone resorption activity.

### Rab32/38 DKO mice exhibited high trabecular bone density

To study the roles of Rab32 and Rab38 *in vivo*, we first examined the locations of these molecules inside bone tissues. Fluorescence immunostaining for Rab32 or Rab38 followed by hematoxylin and eosin (H&E) staining was performed on femur tissue sections prepared from 8-week-old WT mice. Rab32 was detected at the contact areas between the bone and attached cells, and Rab38 appeared in similar areas. Rab32 exhibited significant accumulation in some areas of the bone surface, whereas Rab38 showed a wider distribution within the attached cells (Fig. 5B and C). Both Rab32- and Rab38-positive signals were scarce inside the bone matrix (Fig. 5B and C). These images are consistent with the view that Rab32 and Rab38 play roles in osteoclasts *in vivo*.

**Fig. 6:**
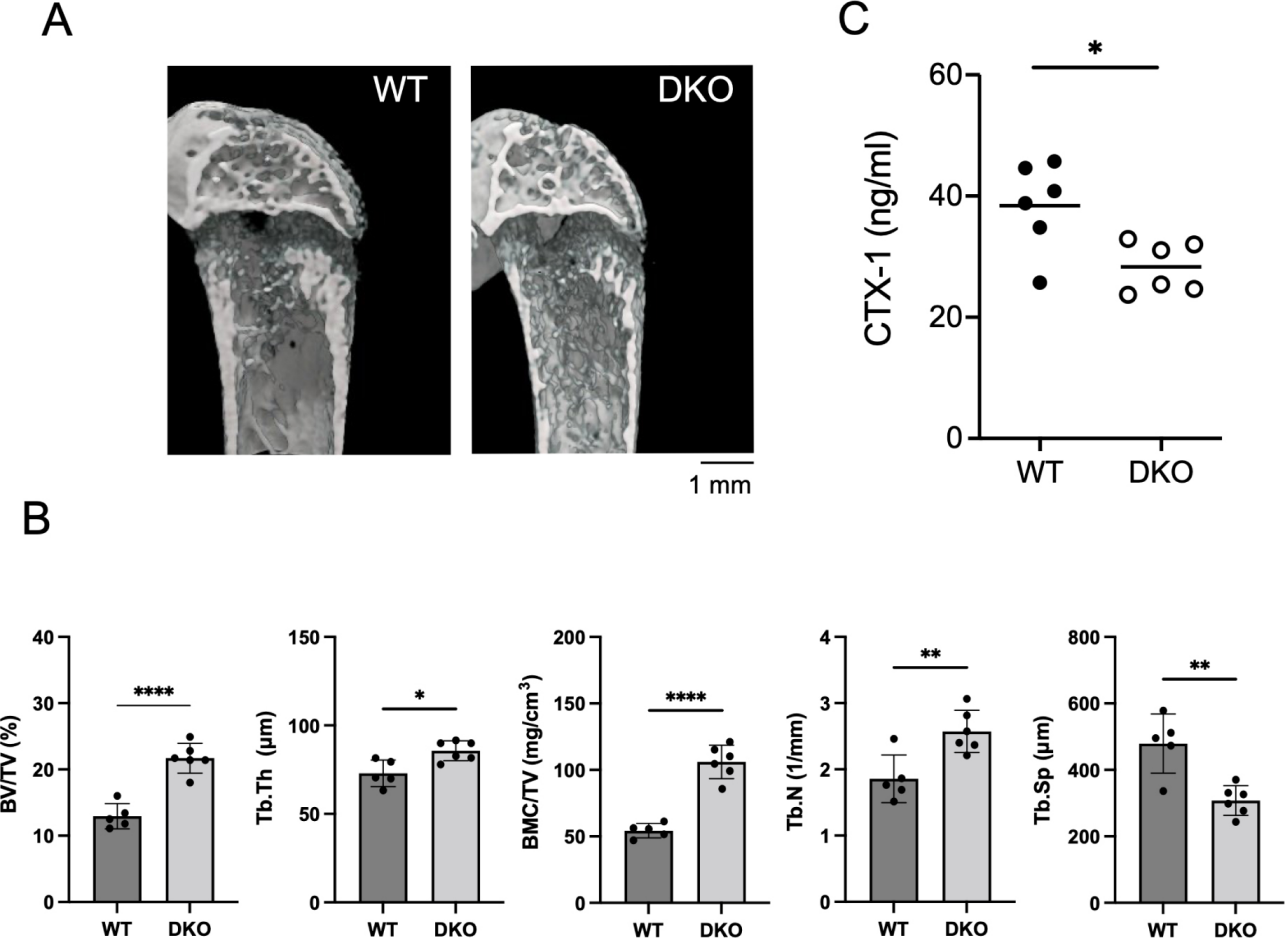
Rab32/38 DKO significantly increased trabecular bone volume in the femur A: 3D micro-CT images of femurs from 8-week-old male WT and Rab32/38 DKO mice. The Rab32/38 DKO mouse has increased trabecular bone volume due to denser bone trabeculae. Scale bar: 1 mm. B: Micro-CT analysis of trabecular bone area in the femurs of 8-week-old male WT and Rab32/38 DKO mice. The trabecular bone structural parameters are as follows: BV/TV (%), bone volume per tissue volume; Tb.Th (μm), trabecular thickness; BMC/TV (mg/cm^3^), bone mineral content per trabecular bone volume; Tb.N (1/mm), trabecular number; Tb.Sp (μm), trabecular separation (n = 6). C: Measurement of CTX-I in sera of 8-week-old WT and Rab32/Rab38 DKO male mice using ELISA. Each dot represents one mouse (B and C). Each data point is shown as mean ± SD. Statistical analysis used the unpaired Student’s *t*-test (* *p*<0.05, ** *p*<0.01, and **** *p*<0.0001).

Next, we analyzed the right femurs of 8-week-old male WT and Rab32/38 DKO mice using micron-scale computed tomography (micro-CT). The 3D reconstituted images of trabecular bone in Rab32/38 DKO mice showed a significantly more dense trabecular bone structure than in WT mice (Fig. 6A). Indeed, we observed increases in four trabecular bone structure parameters, namely bone volume per tissue volume (BV/TV), trabecular thickness (Tb.Th), trabecular number (Tb.N) and bone mineral content per tissue volume (BMC/TV), as well as a decrease in trabecular separation (Tb.Sp), implying that osteogenesis predominates over bone resorption in the trabecular bone of Rab32/38 DKO mice (Fig. 6B). Notably, both Rab32 and Rab38 single KO mice showed a similar trabecular bone phenotype as WT mice (Sup. fig. 3), indicating the complementary role of Rab32 and Rab38 in bone homeostasis. We also used ELISA to measure the serum levels of carboxy-terminal collagen I (CTX-I) crosslinks; these are released as a result of bone resorption and represent osteoclastic activity *in vivo* (Garnero *et al.*, 2003). We found that the serum CTX-I level was significantly lower in Rab32/38 DKO mice than in WT mice (Fig. 6C), indicating that bone resorption is abrogated in Rab32/38 DKO mice *in vivo*.

### Bone deformation occurred in Rab32/38 DKO mice

**Fig. 7:**
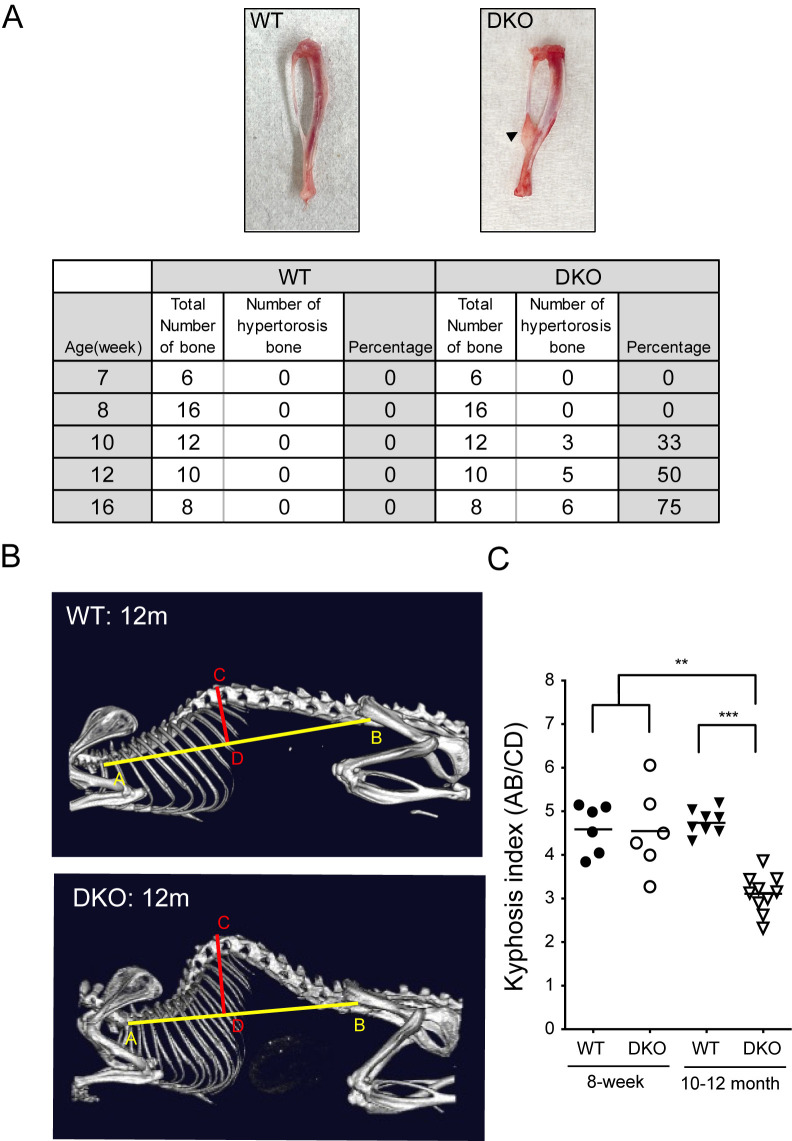
Rab32/Rab38 DKO caused bone deformation A: Images of the fibulas from 16-week-old WT and Rab32/38 DKO mice. The black triangle indicates fibular hyperostosis. The table shows the numbers and percentages of bones with fibular hyperostosis in WT and Rab32/Rab38 DKO male mice at different ages. B: Micro-CT images of the spines of 12-month-old WT and Rab32/38 DKO male mice. C: The graph shows the kyphosis index (KI), defined as the distance AB divided by the distance CD (Laws and Hoey, 2004) from 8-week-old and 10 to 12-month-old WT and Rab32/38 DKO male mice. AB is the distance between the caudal margin of the last cervical vertebra and the caudal margin of the sixth lumbar vertebra. CD is the distance from the dorsal edge of the vertebra at the point of greatest curvature and a line perpendicular to the line AB. Each dot represents the KI of one mouse. Each data point is shown as mean ± SD. Statistical analysis used the unpaired Student’s *t*-test (** *p*<0.01 and *** *p*<0.001).

As described above, there were differences between WT and Rab32/38 DKO mice regarding bone indices at 8 weeks of age, but no clear discrepancy in external appearance. However, while harvesting bone marrow from the femur, we noticed that 10-week-old Rab32/38 DKO male mice exhibited fibular hyperostosis at the talofibular syndesmosis, the distal junction of the tibia and fibula (Fig. 7A). The prevalence of hyperostosis increased with age past 10 weeks (Fig. 7A). This phenotype was not observed in females (data not shown). Furthermore, middle-aged (10- to 12-month-old) Rab32/38 DKO male mice showed increased curvature between the beginning of the first thoracic vertebra and the end of the last lumbar vertebra (Fig. 7B). We histologically examined the corresponding regions, but could not identify differences between WT and DKO mice (data not shown). This kyphosis is usually observed in older WT male mice, usually those aged 24 months (Grunewald *et al.*, 2021). The kyphosis index, representing the degree of curvature (Laws and Hoey, 2004), was shown to be significantly decreased in Rab32/38 DKO male mice compared to WT. This phenotype was also absent in females (data not shown). These results indicate that bone homeostasis is disturbed with increasing age in Rab32/38 DKO male mice.

## Discussion

In the present study, we showed that bone mass was increased in Rab32/38 DKO mice, indicating that bone formation predominates over bone destruction in the balance of bone homeostasis. This is at least partly because of that the bone resorption activity of DKO osteoclasts is decreased. In male mice this results in bone structure abnormalities that become more pronounced with age.

Since Rab38 expression is markedly induced following osteoclast differentiation (Charles *et al.*, 2012; Noda *et al.*, 2023), it is possible that Rab38 affects osteoclast differentiation. However, the expression of specific markers for osteoclast differentiation in osteoclasts derived from Rab32/38 DKO mice did not differ *in vitro* from those derived from WT mice, indicating that Rab32/38 DKO cells can differentiate into multinucleated osteoclasts containing bone resorption enzymes *in vitro*. On the other hand, osteoclasts from Rab32/38 DKO mice had significantly reduced bone resorption capacity *in vitro*. Our results suggest that the loss of bone resorption function resulting from Rab32/38 DKO is due to defective trafficking of TRAP, CTSK, and V-ATPase to the extracellular space and plasma membrane, which together are equivalent to the resorption pit and ruffled border. In addition, actin ring formation was slightly decreased in Rab32/38 DKO osteoclasts, suggesting that bone resorption functionality in the pit area is also affected in Rab32/38 DKO mice. It was reported that osteoclasts exist in one of two states: an active state, involving attachment to the bone surface with subsequent bone resorption, and a resting state, marked by detachment from the bone (Kikuta *et al.*, 2013). As actin ring formation efficiency was lower in DKO mice than in WT mice, osteoclast attachment efficiency may be altered in the former.

In our recently published paper (Noda *et al.*, 2023), we report that Rab32 and Rab38 co-localize to a novel osteoclast-specific LRO. In this study, we show that Rab32 and Rab38 are involved in the expression of osteoclast function. Some Rab32- and Rab38-positive LROs are located in close proximity to resorption pits in osteoclasts, and CTSK and TRAP are contained within the LROs of mononuclear osteoclasts after RANKL stimulation (Noda *et al.*, 2023). The Rab32- and Rab38-positive LRO is also positive for V-ATPase. These LROs are at or near the final stage of trafficking to the ruffled border. Based on these observations, we suggest that Rab32/38 DKO does not support the proper transport of these enzymes, leading to reduced bone resorption. Rab7 and Rab27 are known to be involved in vesicle trafficking in osteoclasts (Shimada-Sugawara *et al.*, 2015; Zhao *et al.*, 2001). In particular, V-ATPase plays a crucial role in recruiting Rab7, together with its GEF Mon1-Ccz1, to this LRO (Matsumoto *et al.*, 2022, 2018), and therefore one possibility is that Rab7 recruitment is disturbed in Rab32/38 DKO osteoclasts. The involvement of these Rab proteins in the Rab32- and Rab38-positive LRO should be clarified in the future.

It has been reported that Rab38 single KO mice do not exhibit bone formation abnormalities (Charles *et al.*, 2012). In this study, we also confirmed that neither Rab32 nor Rab38 single KO mice showed bone phenotypes. It should be noted that there is no difference between the coat color of Rab38 null mutant mice and that of Rab38 point mutated chocolate mice. The remarkable bone-related phenotype of our Rab32/38 DKO mice, but not Rab32 or Rab38 single KO mice, indicates that Rab32 and Rab38 function redundantly in osteoclasts, just as they do in melanosomes and in dense granule biogenesis (Aguilar *et al.*, 2019; Bultema *et al.*, 2014). V-ATPase *a*3 subunit KO mouse had an abnormal bone phenotype but a normal melanosome phenotype, even though *a*3 was localized on melanosomes in WT mice (Matsumoto *et al.*, 2018; Sun-Wada *et al.*, 2009; Tabata *et al.*, 2008). Rab32/38 DKO mice demonstrated both bone- and melanosome-related phenotype abnormalities, clearly establishing the relevance of osteoclasts and melanosomes in terms of LRO function. Rab32 single KO mice did not show an abnormal color phenotype, suggesting that Rab38 possess greater influence over melanosome biogenesis. Rab32 and Rab38 have similar mRNA expression levels after osteoclast differentiation (Noda *et al.*, 2023), so it is reasonable that both are important in osteoclast function. Meanwhile, the lack of an abnormal bone-related phenotype in Rab38 single KO mice suggests that Rab32 alone can fulfill most osteoclast-related functions. Therefore, it remains unclear why Rab38 is acutely induced during osteoclast differentiation, and the specific role of this protein need be further investigated.

Although we could not detect altered femoral length or defective tooth eruption, including impaired feeding ability, in DKO mice, this study revealed abnormal fibular hyperplasia in male mice, possibly caused by an increase in bone mass. Furthermore, middle-aged male mice exhibited extreme kyphosis, which is usually observed in much older male mice (Harkema *et al.*, 2016). Although the cause of this phenotype is not clearly understood, it seems to share some characteristics with the phenotype known as diffuse idiopathic skeletal hyperostosis, a disease that causes bone hyperplasia of the spine, especially in middle-aged and older human males (Kuperus *et al.*, 2020). The kyphotic phenotype that we observed may also be caused by osteoclast dysfunction. The phenotypes characterized by fibular and spinal abnormalities in Rab32/38 DKO mice were observed only in males and not in females. It is conceivable that bone loss progresses with age in C57BL/6J mice just like in humans (Ferguson *et al.*, 2003; Halloran *et al.*, 2002), and its magnitude is influenced by gender (Mumtaz *et al.*, 2020). Sex hormones probably also play a role, since they affect osteoporosis and other bone-related phenomena as well (Almeida *et al.*, 2017). For example, it is known that bone mass is generally higher in male mice due to the activation of osteoblasts via androgen receptors (Kawano *et al.*, 2003). This may be the reason why only males showed these bone related phenotypes, assuming that osteoclast function was equally impaired in both male and female Rab32/38 DKO mice. Although in this study, we did not measure bone mass in female mice in order to avoid the effect of estrogen on osteoclast, more controlled study should be followed to pursue this point.

In this study, we revealed that Rab32 and Rab38 are crucial for proper bone formation. Our results indicate that both Rab32 and Rab38 may serve as drug targets for suppressing osteoclast function and may be developed like a bisphosphonate or like odanacatib, a CTSK inhibitor, as a therapeutic agent for rheumatoid arthritis, osteoporosis, and periodontitis.

## Materials and Methods

### Mice

C57BL/6J mice were purchased from Japan SLC (Shizuoka, Japan). All mice were maintained under specific pathogen-free conditions in the animal facility of the Graduate School of Dentistry, Osaka University. Mice used for isolation of bone marrow cells and collection of femurs, tibias, and fibulas, were appropriately euthanized using carbon dioxide inhalation. Mice used for whole blood collection and micro-CT imaging were euthanized using a mixture of three anesthetics (3% Domitor (medetomidine hydrochloride 1.0 mg/mL, AHD1, ZENOAQ, Koriyama, Japan), 8% Dormicum (midazolam 5.0 mg/mL, 17008A2, Astellas Pharma, Tokyo, Japan), and 10% Betolfal (butorphanol tartrate 5.0 mg/mL, VETLI5, Meiji Seika Pharma, Tokyo, Japan) in MilliQ water). This mixture was administered intraperitoneally at a dose of 0.1 mL per 10 g of body weight. All animal studies were approved by the Institutional Animal Experiments Committee of Osaka University Graduate School of Dentistry (29-008-0) and the Gene Modification Experiments Safety Committee of Osaka University.

### Generation of Rab32/38 DKO mice

The CRISPR/Cas9 system was injected into mouse embryos by electroporation at the Institute of Experimental Animal Sciences, Osaka University Medical School, as previously reported (Kaneko and Mashimo, 2015). The guide RNAs were designed to target the Rab32 exon 1 and Rab38 exon 1 loci (Fig. 1A). F_0_ mosaic mutant mice were crossed with C57BL/6J mice to produce heterozygous mice with a 4-nucleotide base-pair deletion in Rab32 and a 5-nucleotide base-pair deletion in Rab38 in F_1_ mice. Generation of F_1_ mice was confirmed by Sanger sequencing, with DNA collected from mouse ears used as a PCR template. Primers used for PCR and Sanger sequence analysis are as follows. Rab32: GGTAGCCAGGGAAGAGGAAG (Forward) and ACCGGGAGACAGAGAGGAGT (Reverse), Rab38: GCACCAGCTCCCTATCCTG (Forward) and ACTCCTCACTGGCTCACTCC (Reverse). The resulting F_1_ heterozygous mutant mice were crossed with WT mice to obtain F_2_ heterozygous mutant mice. Furthermore, F_1_ heterozygous mutant mice were crossed with F_2_ heterozygous mutant mice to obtain F_3_ homozygous mutant mice. Homozygous mutant mice were then crossed with each other to maintain the Rab32/38 DKO mouse line.

### Genotyping of Rab32/38 DKO mice

DNA was extracted from mouse ear specimens using the KAPA Express Extract Kit (Roche, Basel, Switzerland) and purified by isopropanol precipitation. PCR was performed using Tks Gflex DNA Polymerase (Takara Bio, Kusatsu, Japan) with initial denaturation at 94°C for 1 min, followed by 40 cycles of denaturation at 98°C for 10 s, annealing at 60°C for 15 s, and elongation at 68°C for 30 s. The resulting PCR products were mixed with the gel loading dye. Generation of 15% polyacrylamide gel was performed by mixing 40(w/v)%-acrylamide/bis mixed solution (19:1) (Nacalai Tesque, Kyoto, Japan), 5 X TBE (44.5 mM Tris-borate (pH 8.3), 10 mM EDTA), 10% ammonium persulfate, and TEMED, and poured in a slab gel plate. After electrophoresis using 1 X TBE as buffer at 40 mA for 2 h, the polyacrylamide gel was immersed in a 0.5 μg/mL ethidium bromide solution for 10 min and detected using DigiDoc-It Darkroom (UVP). The following primers were used for genotyping.

Rab32: CGAGGGACTAGGGCAACAG (Forward) and AAAGAGGTGCTCTCGGGTC (Reverse), Rab38: ACAAGGAGCACCTGTACAAG (Forward) and AGCGCTTGATAATGCTGGTC (Reverse).

### BMM isolation and culture

Male C57BL6J mice or Rab32/38 DKO mice between 7 and 10 weeks of age were used for the experiments. Under aseptic conditions, the femurs and tibias of mice were harvested and the medullary cavity of the bones were rinsed with α-MEM with L-glutamine and phenol red (135-15175, Fujifilm Wako Pure Chemical, Osaka, Japan) containing 60 μg/mL kanamycin (Fujifilm Wako Pure Chemical) in a 5-mL syringe and centrifuged at 250 × *g* for 5 min. Red blood cell lysing buffer (150 mM NH_4_Cl, 10 mM KHCO_3_, 0.1 mM EDTA (pH 7.4)) was added to remove blood cells, and the remaining cells were used as bone marrow cells. These cells were suspended in 10 mL of α-MEM basal medium (α-MEM with L-glutamine and phenol red containing 60 μg/mL kanamycin and 10% FBS), seeded into 100-mm petri dishes, and cultured in 5% CO_2_ at 37°C overnight. As the M-CSF conditioned medium, the culture supernatant of CMG14-12 cells, a mouse M-CSF-producing Ltk^-^ cell line, was collected in α-MEM basal medium (Takeshita *et al.*, 2000). After 10-h incubation, non-adherent cells in the supernatant were collected and seeded in 24-well plates (2.5 × 10^5^ cells/well), 48-well plates (1.0 × 10^5^ cells/well), or 96-well plates (4.0 × 10^4^ cells/well) in α-MEM basal medium with 10% M-CSF conditioned medium. After 48-h culture, adherent cells were defined as BMMs and were replaced with differentiation medium (α-MEM basal medium, 2% M-CSF conditioned medium, and 400 ng/ml GST-RANKL, prepared as previously described (Kimura *et al.*, 2015)) to induce differentiation into osteoclasts. This point was designated as culture day 0. After 3 days, half of the medium was replaced and osteoclasts were collected at 5 or 6 days of differentiation (Greenblatt *et al.*, 2015; Mizoguchi *et al.*, 2009).

### TRAP staining

TRAP staining was performed using a TRAP Staining Kit (AK04F, Cosmo Bio, Tokyo, Japan). Bone marrow derived from the femurs and tibias of WT and Rab32/38 DKO mice was harvested in 96-well plates and cultured in α-MEM basal medium with M-CSF for 2 days to induce BMMs. Then, RANKL was added and culture was performed for 5 days to induce differentiation into osteoclasts. Osteoclasts on 96-well plates were fixed in 10% Formalin Neutral Buffer Solution and incubated with Chromogenic Substrate Solution at 37°C for 20–60 min. Once sufficiently colored, the reaction was stopped by washing the wells with pure water. TRAP-positive multinucleated cells (three or more nuclei) were counted as differentiated osteoclasts.

### Protein extraction and western blotting

Osteoclast differentiation was induced in differentiation medium for 5 or 6 days in 24-well plates. Cells were then washed with phosphate-buffered saline (PBS) (137 mM NaCl, 2.7 mM KCl, 10 mM Na_2_HPO_4_, 1.76 mM KH_2_PO_4_ (pH 7.4)), and 50 μL lysis buffer (50 mM Tris-HCl (pH 7.5), 150 mM NaCl, 1% TritonX-100, EDTA-free Protease Inhibitor Cocktail Tablets (Roche)) was added to each well. After the plates were allowed to stand for 10 min on ice, the whole-cell lysate was collected, and 6 wells were combined into one sample. After centrifugation at 20,400 × *g* for 10 min, supernatants were collected and then protein quantification was performed by Pierce Coomassie Plus (Bradford) Assay Kit (Thermo Fisher Scientific, Waltham, MA, USA). Protein concentrations were adjusted to 1 mg/mL.

For protein extracts from mouse organs, small samples of tissue were washed in PBS and placed in one well in the 6-well plate containing 1 mL lysis buffer, and stainless mesh (40 mesh/inch, E9111, Q-ho Metal Works, Osaka, Japan) was placed over the tissue on ice and pushed through it using a 5-mL syringe plunger head. The whole-tissue lysate was collected into 1.5 mL tubes, stored at –80°C overnight, and then stored at 4°C until totally thawed. This freeze-and-thaw step was repeated twice, and protein extracts were collected after centrifugation as described above. Protein concentrations were adjusted to 5 mg/mL.

The samples were suspended in sample buffer (300 mM Tris-HCl (pH 6.8), 12% SDS, 30% glycerol, 0.006% bromophenol blue, 0.6 M 2-mercaptoethanol) and boiled for 3 min. Ten micrograms of protein from cell samples or 50 μg of protein from tissue samples were electrophoresed in SDS-PAGE using 15% polyacrylamide gel and running buffer (25 mM Tris-HCl (pH 8.3), 191 mM glycine, 0.1% SDS). The proteins were transferred to polyvinylidenefluoride (PVDF) membranes (GE Healthcare, Chicago, IL, USA) using transfer buffer (25 mM Tris-HCl (pH 8.3), 192 mM glycine, 20% methanol) in a transfer device (NA-150: Nihon Eidoh, Tokyo, Japan) (100 V, 150 mA, 1 hr). PVDF membranes were treated with PBS-T (3.2 mM Na_2_HPO_4_, 0.5 mM KH_2_PO_4_, 1.3 mM KCl, 135 mM NaCl, 0.05% Tween 20 (pH 7.4)) containing 5% skim milk (Morinaga Milk Industry, Tokyo, Japan) for 60 min at room temperature for blocking. The primary antibody was reacted with a diluted solution of Can Get Signal (TOYOBO, Osaka, Japan) Solution I for 1 h at room temperature, followed by washing with PBS-T for 15 min. Then, the secondary antibody was reacted with a solution diluted with Can Get Signal (TOYOBO) Solution II for 1 h at room temperature, followed by washing in PBS-T for 15 min. After washing, the membrane was reacted with ECL Select Western Blotting Detection Reagent (GE Healthcare), and bands were detected using Gene Gnome 5 (Syngene, Cambridge, UK). The dilution ratio of each antibody was as follows: primary antibodies were anti-Rab32 rabbit (Ohbayashi *et al.*, 2012): 1/1000, anti-Rab38 rabbit (Ohbayashi *et al.*, 2012): 1/1000, anti-cathepsin K (sc-48353, E-7, Santa Cruz Biochemistry, Dallas, TX, USA): 1/200, and anti-GAPDH (2275-PC-100, R&D System, Minneapolis, MN, USA): 1/5000; secondary antibodies were anti-rabbit IgG HRP (Cell Signaling Technology, Danvers, MA, USA): 1/2000 and anti-mouse IgG HRP (SouthernBiotech, Birmingham, AL, USA): 1/10000.

### Immunofluorescence staining of osteoclasts and microscopy

For staining the V-ATPase *a*3 subunit, 0.4% saponin (S0019-25G, Tokyo Chemical Industry, Tokyo, Japan) was used for permeabilization and 5% normal donkey serum (017-000-121, Jackson ImmunoResearch, West Grove, PA, USA) was used for blocking. Prepared samples were then incubated with the primary antibody (chicken anti-*a*3) 1/500 (Sun-Wada *et al.*, 2011) diluted in blocking buffer (0.4% saponin (S0019-25G, Tokyo Chemical Industry), 5% normal donkey serum (017-000-121, Jackson ImmunoResearch), 1% FBS (F7524, Sigma-Aldrich, St. Louis, MO, USA) in PBS) for 4°C overnight. After washing three times with blocking buffer, samples were incubated with the following secondary antibodies for 1 h at room temperature: Alexa Fluor 488–AffiniPure Donkey Anti-Chicken IgY (IgG) (H + L) 1/500 , Acti-stain 555 Phalloidin (Cytoskeleton, Denver, CO, USA) 1/500, and DAPI (Sigma-Aldrich) 1/1000 diluted in blocking buffer. After washes, a cover glass was mounted on a slide glass with 5 μL of mounting medium (3.8 mM Mowiol 4-888 (81381, Sigma-Aldrich), 3.3 M non-fluorescent glycerol (075-04751, Fujifilm Wako Pure Chemical), 0.2 M Tris-HCl (pH 8.5), 2.5% 1.4-diazabicyclo[2.2.2]octane (D27802, Sigma-Aldrich)) for 1 h at room temperature or 4°C overnight. Samples were observed under a confocal laser scanning microscope TSC SP8 (Leica, Wetzlar, Germany). The objective lens used was HC PL APO CS2 63 × 1.40 OIL (Leica).

### Quantitative PCR

Total RNA of BMMs and osteoclasts cultured in 24-well plates was extracted using TRIsure (Bioline, Memphis, TN, USA). The extracted RNA was used as a template for reverse transcription using the iScript cDNA Synthesis Kit (Bio-Rad, Hercules, CA, USA) to generate complementary strand DNA (cDNA). Real-time PCR analysis was performed using QuantiTect SYBR Green PCR (Qiagen, Helden, Germany), with the following cDNA used as templates and primers for genes specific for osteoclast differentiation. GAPDH: AAATGGTGAAGGTCGGTGTG (Forward), TGAAGGGGTCGTTGATGG (Reverse), CTSK: GCCAGGATGAAAGTTGTATG (Forward) CAGGCGTTGTTCTTATTCC (Reverse), TRAP: GCTGGAAACCATGATCACCT (Forward) GAGTTGCCACACAGCATCAC (Reverse), DC-STAMP: TGTATCGGCTCATCTCCTCCAT (Forward) GACTCCTTGGGTTCCTTGCTT (Reverse). The Step One Plus Real-time PCR System (Applied Biosystems, Waltham, MA, USA) was used for reaction and detection. Relative gene expression levels for each gene were calculated according to the ∆∆Ct method (Livak and Schmittgen, 2001), using GAPDH as the internal control gene. The fold induction of osteoclast marker genes was normalized to the basal levels in BMMs, and then the fold induction of mRNA was compared between WT and Rab32/38 DKO osteoclasts.

### Bone resorption assay

Bone resorption activity was evaluated using a Bone Resorption Assay Kit (BRA-24kit, PG Research, Tokyo, Japan). Bone marrow derived from femurs and tibias of WT and Rab32/38 DKO mice was cultured on a FACS/calcium phosphate-coated 48-well plate in α-MEM basal medium with M-CSF for 2 days to induce BMMs. Then, BMMs were induced to differentiate into osteoclasts using differentiation medium without phenol red for 5 days, and the culture supernatant was collected. The fluorescence intensity of the collected supernatant was measured at an excitation wavelength of 485 nm and an emission wavelength of 535 nm by Twinkle LB 970 (Berthold, Bad Wildbad, Germany), a microplate reader. For pit area analysis, each well was treated with 2.5% sodium hypochlorite for 5 min to remove cells, then the wells were washed with distilled water and dried. Each well was randomly photographed under an optical microscope with a 10x objective, and the total pit area per image was measured using ImageJ software. In brief, we drew the outline of each pit area and measured the number of pixels per area. All pixels from a single image were summed up, and six images per well and three wells per condition were analyzed, with results transformed into an area unit (μm^2^) using the scale in a hemocytometer as reference.

### TRAP activity assay

Osteoclasts were prepared in 96-well plates by phenol-red free differentiation medium. The culture supernatants were collected and diluted 5-fold by TRAP activity assay buffer (TAAB) (1 mM ascorbic acid, 0.1 mM FeSO_4_-7H_2_O, 0.1 mM (NH_4_)_2_SO_4_, 0.1 M sodium acetate, 0.15 M KCl, 10 mM sodium (+)-tartrate dihydrate, 0.1% Triton-X100 (pH 5.8)). Cell pellets were washed twice by PBS, lysed by 100 μL TAAB on ice for 10 min, and further diluted 10-fold in TAAB buffer. Ten μL of TRAP substrate (100 mM p-nitrophenylphosphoric acid disodium salt (25019-52, Nacalai Tesque) dissolved in TAAB) was added into 200 μL of diluted supernatant or cell lysate and incubated for 10 min at 37°C, then 50 μL of 0.5 M NaOH was added to stop the reaction. Absorbance at 405 nm was measured by iMark (Bio-Rad). The assay background was cell-free medium or TAAB buffer.

### CTSK activity assay

CTSK activity in cell culture supernatants and cell lysates was detected by Magic Red-Cathepsin K (#6135, ImmunoChemistry Technologies, Davis, CA, USA). Osteoclasts were prepared with phenol-red free differentiation medium in 24-well plates. To collect CTSK, 300 μL fresh differentiation medium was directly added into each well every 3 days without removing culture medium. Cell supernatants were collected 9 days after differentiation induction, and cell pellets were washed twice with PBS and lysed with 1% Triton-X100, 150 mM NaCl, 50 mM Tris-HCl (pH 7.8). Magic Red staining solution in DMSO was diluted 10-fold in H_2_O and 8 μL was added to 200 μL of supernatant or cell lysate. After 15-min incubation at room temperature, fluorescence signals (Ex. 530 nm, Em. 590 nm) were detected by the PowerScan HT fluorescence microplate reader (BioTek, Winooski, VT, USA). Medium or lysis buffer was used as background control.

### 3D micro-CT and bone analysis

For 3D analysis of bone, the distal end of the femur of an 8-week-old male mouse was used to acquire images using a 3D micro-CT scanner R_mCT2 (Rigaku, Tokyo, Japan) with the X-ray source conditions set to 90 kV voltage, 160 μA current, and FOV of 10. The 4-mm section of trabecular bone starting at 0.2 mm from the distal growth plate was reconstructed three dimensionally using TRI/3D-Bone software (RATOC Systems Engineering, Tokyo, Japan). Bone density calibration was performed using a bone mineral density (BMD) phantom (1202-63, Ratoc). The BMD phantom was scanned under the same conditions as those used to analyze femurs, and a BMD calibration curve was created using CT values. According to the manufacturer’s instructions, we measured the femurs with extraction level thresholds ranging from 550 to 600 for cortical bone extraction and 200 to 300 for trabecular bone extraction, and maximum extraction width thresholds were used for both cortical and trabecular bone extraction. The following parameters were analyzed: bone volume per tissue volume (BV/TV (%)), trabecular thickness (Tb.Th (μm)), bone mineral content per tissue volume (BMC/TV (mg/cm^3^)), number of trabecular bone (Tb.N (1/mm)) and trabecular separation (Tb.Sp (μm)).

Mouse fibulas were analyzed using 7- to 16-week-old male mice as indicated in Fig. 7A, and spines were analyzed using 8-week-old or 10- to 12-month-old male mice. A fibula was collected and evaluated by counting the presence or absence of fibular hyperostosis. For spine kyphosis analysis, a 3D micro-CT scanner was used to acquire images of the cervical to thoracic spine after mice were completely anesthetized. Scanning conditions were as follows: FOV, 73; acquisition time, fine 2 min. The distance AB was defined as that between the caudal margin of the last cervical vertebra and the caudal margin of the sixth lumbar vertebra. The distance CD was defined as that from the dorsal edge of the vertebra at the point of greatest curvature to a point perpendicular to the line AB. The kyphosis index (KI) was the distance AB divided by the distance CD (Laws and Hoey, 2004).

### Detection of serum C-terminal telopeptides of type I collagen (CTX-I)

Whole blood was collected from 8-week-old WT and Rab32/Rab38 DKO male mice under regular feeding conditions. The blood was placed at room temperature for 30 min and then centrifuged at 16,300 × *g* at 4°C for 10 min. The supernatants were collected and used immediately or stored at –80°C. CTX-I in 20 μL serum per test were detected by a Rat-Laps (CTX-I) EIA kit (AC-06F1, Immunodiagnostic Systems, Tyne and Wear, UK). In the final step, the absorbance at 450 nm and the reference absorbance at 570 nm were measured by iMark (Bio-Rad).

### H&E and immunofluorescence staining of mouse bone

Fluorescence immunostaining and H&E staining and image superimposition were performed as previously reported (Usami *et al.*, 2019). Femurs were harvested from 8-week-old male WT mice, fixed in 4% paraformaldehyde (PFA) at 4°C overnight, and demineralized in 10% EDTA (pH 7.0) for 14 days. Demineralized bone tissue was embedded in paraffin and sectioned at 4-μm thickness using a Leica CM1850 (Leica). Formalin-fixed, paraffin-embedded (FFPE) sections were mounted on glass slides and dried at 40°C overnight. Then, the FFPE sections were baked for 1 h at 60°C. Paraffin was then removed from the FFPE sections using xylene and ethanol sequentially (100%, 100%, 90%, 80%, 70%, each 10 min) using ASP300S (Leica), and rinsed in water for rehydration. To block endogenous peroxidase activity, the tissue sections were soaked with 3% H_2_O_2_ in methanol for 10 min. To block nonspecific binding, tissue sections were incubated in 5% BSA and 1% goat serum in PBS for 30 min at room temperature. After that, sections were incubated with primary antibody (diluted in 1% BSA in PBS) at 4°C overnight. On the next day, the secondary antibody was treated at room temperature for 1 h. Finally, mounting medium was added to the slide and topped with a coverslip. The dilution ratio of each antibody is as follows: primary antibodies were anti-Rab32 rabbit (Ohbayashi *et al.*, 2012): 1/500 and anti-Rab38 rabbit (Ohbayashi *et al.*, 2012): 1/500; the secondary antibodies was Alexa Fluor 488-conjugated anti-rabbit (Thermo Fisher). The coverslip of the section was gently removed by soaking in PBS followed by standard hematoxylin and eosin (H&E) staining (Cardiff *et al.*, 2014). Adobe Photoshop software (CS6, Adobe Systems, San Jose, CA, USA) was used to superimpose the histochemical staining image (×400; DS-Ri2, Nikon, Tokyo, Japan) onto fluorescence images (TSC SP8, Leica).

### Statistical analysis

Data were analyzed with Graph Pad Prism version 7.04. All data are expressed as mean ± standard deviation (SD). Data comparisons between two groups were analyzed using the unpaired Student *t*-test; *p*-values less than 0.05 were considered statistically significant. * *p*<0.05, ** *p*<0.01, *** *p*<0.005, and **** *p*<0.0001.

## Data Availability

The supporting information for this article is available in J-STAGE Data.

## Abbreviations

BMM: bone marrow-derived macrophage
CTSK: cathepsin K
FBS: fetal bovine serum
GAPDH: glyceraldehyde-3-phosphate dehydrogenase
H&E: hematoxylin and eosin
HPS: Hermansky-Pudlak syndrome
LRO: lysosome-related organelle
M-CSF: macrophage colony-stimulating factor
NFATc1: nuclear factor of activated T cells c1
RANKL: receptor activator of NF-κB ligand
CTX-I: cross-linked C-telopeptide of type I collagen
TRAP: tartrate-resistant acid phosphatase
α-MEM: α-minimum essential medium
micro-CT: micro-computed tomography
