# Supplementary material for: Rab32 and Rab38 maintain bone homeostasis by regulating intracellular traffic in osteoclasts
Source: Cell Struct Funct. 2023 Oct 4;48(2):223–39. doi: 10.1247/csf.23061 (PMC11496785; doi:10.1247/csf.23061)
Supplement: Supplementary file 1 — Supplementary Figures [file csf_48_23061_1.zip › 48_23061_1.pdf]

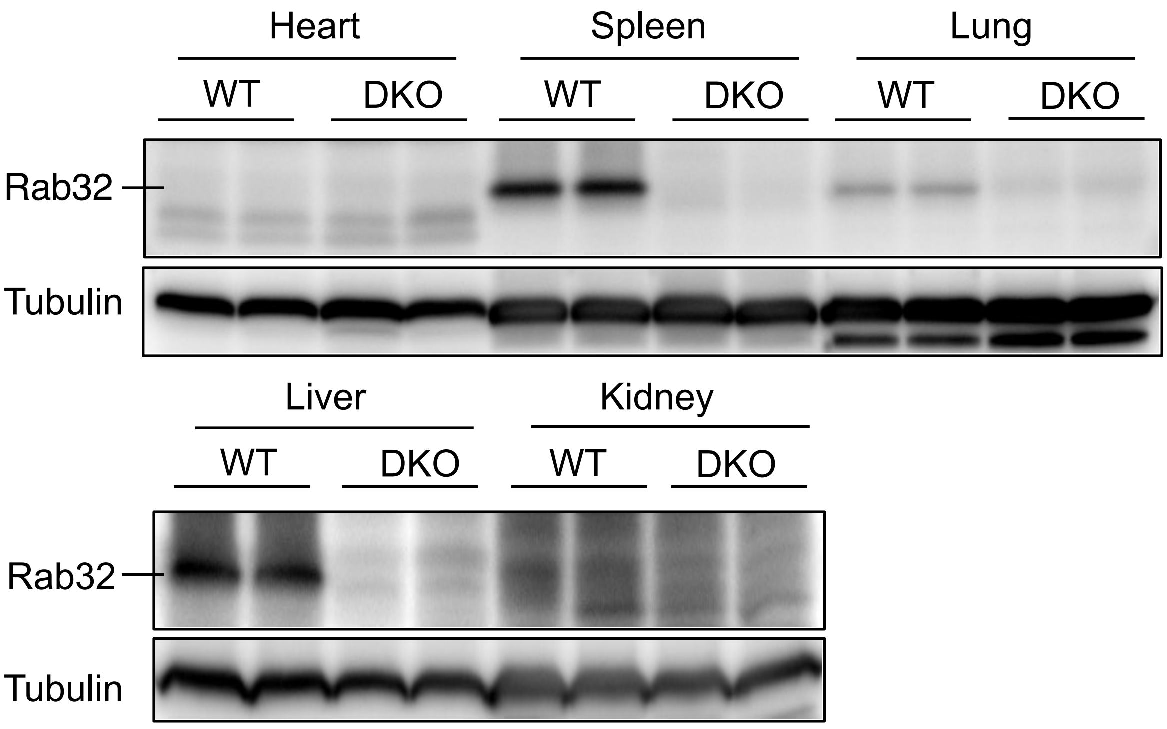

Supplemental figure 1 Rab32 protein expression in mouse organs.

Two WT or Rab32/38 DKO mice were sacrificed at 8 weeks of age, and small samples of organs, including heart, spleen, lung, liver, and kidney, were prepared for western blot analysis using the indicated antibodies.
