# Supplementary material for: Rab32 and Rab38 maintain bone homeostasis by regulating intracellular traffic in osteoclasts
Source: Cell Struct Funct. 2023 Oct 4;48(2):223–39. doi: 10.1247/csf.23061 (PMC11496785; doi:10.1247/csf.23061)
Supplement: Supplementary file 1 — Supplementary Figures [file csf_48_23061_1.zip › 48_23061_2.pdf]

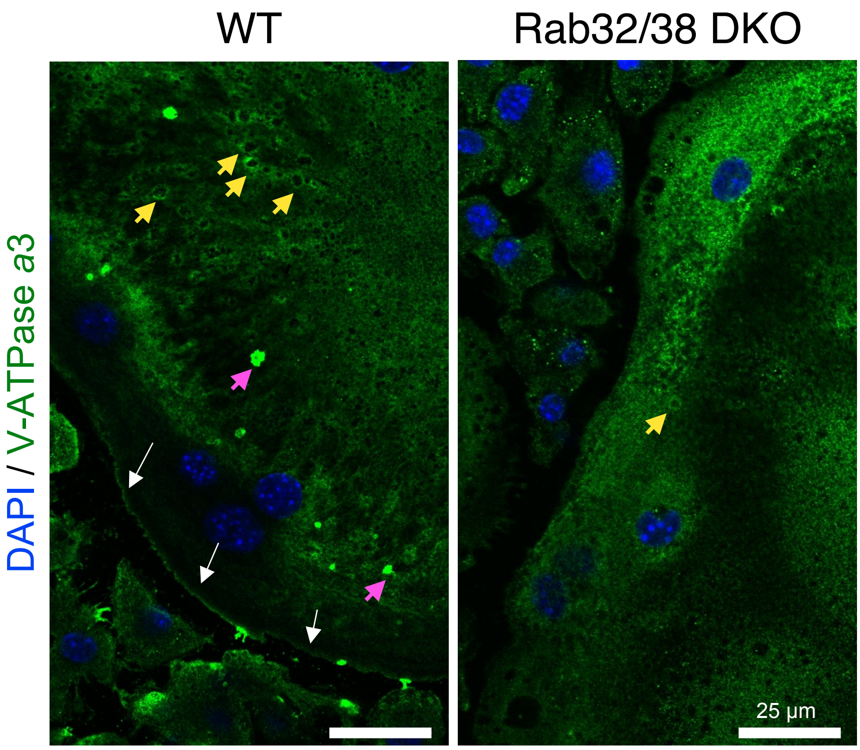

Supplemental figure 2 Accumulation of the V-ATPase *a*3 subunit on the plasma membrane was absent in Rab32/38 DKO osteoclasts.

Immunofluorescence staining images of WT and Rab32/38 DKO osteoclasts. Osteoclasts were fixed with 4% PFA and stained with chicken anti-*a*3 antibodies and DAPI. The green indicates *a*3 subunits (Alexa-488) and the blue indicates DAPI. The plasma membrane is indicated by white arrowheads, *a*3-positive ring structures are indicated by magenta arrowheads, and *a*3-positive intense puncta are indicated by yellow arrowheads. Scale bar: 25 µm.
