# Supplementary material for: Rab32 and Rab38 maintain bone homeostasis by regulating intracellular traffic in osteoclasts
Source: Cell Struct Funct. 2023 Oct 4;48(2):223–39. doi: 10.1247/csf.23061 (PMC11496785; doi:10.1247/csf.23061)
Supplement: Supplementary file 1 — Supplementary Figures [file csf_48_23061_1.zip › 48_23061_3.pdf]

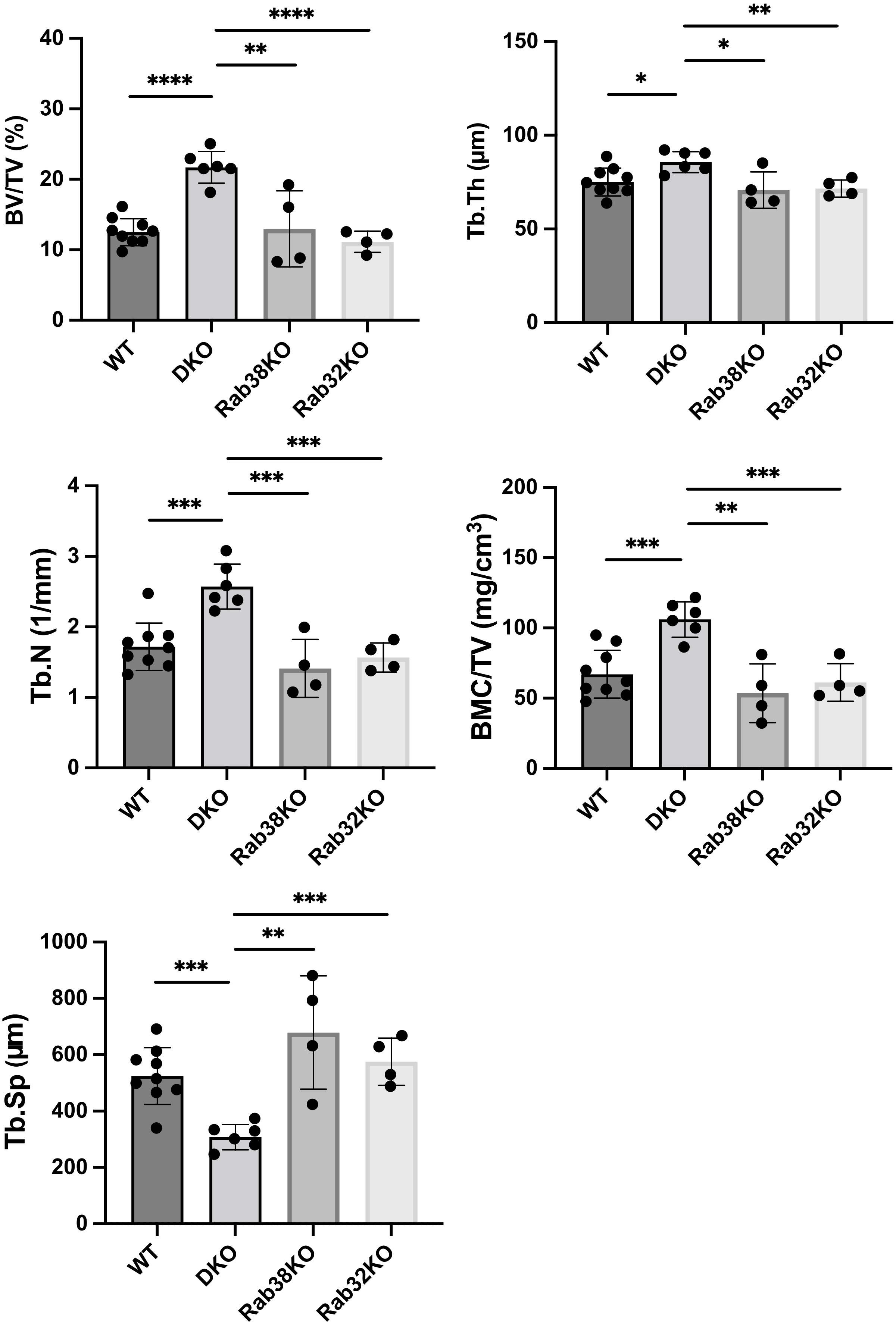

Supplemental figure 3 Neither Rab32 nor Rab38 single KO affected trabecular bone volume in the femur. Micro-CT analysis of trabecular bone area in the femurs of 8-week-old male WT, Rab32 KO, Rab38 KO and Rab32/38 DKO mice. The trabecular bone structural parameters are as follows: BV/TV (%), bone volume per tissue volume; Tb.Th (μm), trabecular thickness; BMC/TV (mg/cm3), bone mineral content per trabecular bone volume; Tb.N (1/mm), number of trabecular bones; Tb.Sp (μm), and trabecular separation (n=4). Rab32 and Rab38 single KO mice show similar bone intensity as the WT group. Each data point represents one mouse, and the graph are shown as mean ± SD. Statistical analysis was performed using the unpaired Student's t-test (* *p*<0.05, ** *p*<0.01, *** *p*<0.001 and **** *p*<0.0001).
